# Effectiveness screening of SARS-CoV-2 (COVID-19) in the municipality of Andratx (Balearic Islands, Spain)

**DOI:** 10.3389/fpubh.2025.1461436

**Published:** 2025-04-23

**Authors:** Margalida Monserrat-Mesquida, Maria Magdalena Quetglas-Llabrés, Cristina Bouzas, Silvia García, Patricia Font, Emma Argelich, Teresa Martínez, Antoni Sureda, Josep A. Tur

**Affiliations:** ^1^Research Group on Community Nutrition and Oxidative Stress, University of the Balearic Islands-IUNICS, Palma de Mallorca, Spain; ^2^CIBEROBN (Physiopathology of Obesity and Nutrition), Instituto de Salud Carlos III, Madrid, Spain; ^3^Health Research Institute of Balearic Islands (IdISBa), Palma de Mallorca, Spain; ^4^Centre de Salut Ponent, IBSalut, Andratx, Spain

**Keywords:** COVID-19, RT-PCR test, immunoglobulin G, immunoglobulin M, SARS-CoV-2 virus

## Abstract

**Background:**

The coronavirus disease 2019 (COVID-19) pandemic was a global health emergency that significantly affected both the wellbeing of individuals and the global economy.

**Objective:**

The aim of this study was to evaluate the degree of SARS-CoV-2 involvement, viral load, and immunological response in adults aged 18–65 who performed essential tasks in the municipality of Andratx (Balearic Islands, Spain) compared to those who did not. Additionally, the study examined these factors in children aged 2–18 years from both groups, if there were any.

**Materials:**

Both groups were monitored between July 2020 and February 2021, in which the degree of involvement, the viral load, and the immunological response to the SARS-CoV-2 were analyzed using questionnaires, polymerase chain reaction (PCR) tests, and ELISA serology tests.

**Results:**

A positive case of RT-PCR test was found in screening the general population. The highest 2019-nCoV(N)-Ig antibody levels in plasma were measured from 1 to 17 February 2021, with the following percentage of positives: 6.8% of essential workers, 9.5% of essential workers’ sons, 7.3% of non-essential workers, and 2.2% of non-essential workers’ sons. However, an increase in levels of anti-SARS-CoV-2 (N) immunoglobulin G (IgG) and anti-SARS-CoV-2 (N) immunoglobulin M (IgM) were produced in session 3, from 9 to 25 November 2020, in both cases in non-essential workers, with a mean of 218.1 ng/mL of anti-SARS-CoV-2 (N) IgG and 31.3 ng/mL of anti-SARS-CoV-2 (N) IgM.

**Conclusion:**

The control measures taken to manage the COVID-19 pandemic in the municipality of Andratx, Mallorca, Spain, were effective.

## 1 Introduction

The coronavirus disease 2019 (COVID-19) pandemic is an infectious disease caused by the respiratory syndrome coronavirus 2 (SARS-CoV-2) (1). It originated in December 2019 in the city of Wuhan (People’s Republic of China) (2), and quickly became a global health emergency and crisis. The pandemic significantly affected both the wellbeing of individuals and the global economy (3).

As of 2 June 2024, a total of 775,583,309 people worldwide had been diagnosed with COVID-19, with 7,050,691 fatalities reported (4). Globally, between 29 April and 26 May 2024, 94 countries reported COVID-19 cases, and 28 countries reported COVID-19 deaths. Based on available data, reported cases and deaths decreased during this period, with over 129,000 new cases and more than 1,800 new deaths, representing an 11 and 36% decrease, respectively, compared to the previous 28 days (1 to 28 April 2024) (5). As of 21 February 2021, the global total of COVID-19 was 110.7 million cases and over 2.4 million deaths since the pandemic began (6).

As of 19 June 2020, there were 8,385,440 confirmed cases of COVID-19 and 450,686 confirmed deaths, affecting 216 countries and territories (7). From 31 December 2019 to 9 June 2020, European countries and the United Kingdom reported 1,444,710 cases (20% of global cases) and 169,207 deaths (42% of global deaths) (8). Spain remains one of the European countries most severely affected by the ongoing COVID-19 pandemic, with over 249,000 confirmed cases and more than 28,000 deaths as of 2 July 2020 (9).

Clinical manifestations of COVID-19 range from mild to severe or fatal. The most common symptoms were non-specific, primarily including fever, cough, and muscle aches (2). Other less common symptoms included sore throat, headache, chills, nausea or vomiting, diarrhea, loss of taste, and eye congestion (10). COVID-19 was clinically categorized into three stages: mild to moderate disease (non-pneumonia and pneumonia), severe disease (characterized by dyspnea, a respiratory rate over 30 breaths/min, and/or oxygen saturation below 93%), and critical disease (marked by respiratory failure, septic shock, and/or multi-organ dysfunction/failure) (11). Many older adult patients with severe illness had underlying chronic conditions, such as cardiovascular disease, lung disease, kidney disease, or cancer (12).

Over 30% of individuals who contract COVID-19, including those without symptoms, and approximately 80% of those hospitalized for the disease may suffer from post-COVID-19 sequelae. This condition, often called “long COVID,” includes persistent symptoms such as fatigue and cognitive impairment, along with other lasting neuropsychiatric issues (e.g., depression) and physical problems (e.g., shortness of breath) (13).

Regarding testing, real-time reverse transcription-PCR (RT-PCR) was established as the gold standard for the early detection of viruses and the most widely used method for identifying SARS-CoV-2 (14, 15). Immunological responses take longer to manifest, with antibodies typically starting to appear approximately 6 days after symptoms begin, coinciding with a decline in viral RNA levels (16).

The coronavirus family includes four structural proteins: envelope (E), membrane (M), nucleocapsid (N), and spike (S) proteins. Two of these proteins are particularly important antigenic sites for the development of COVID-19 serological assays. Most serological methods have concentrated on detecting serum antibodies against the coronavirus S proteins and N proteins (17), as these have shown the highest sensitivity among commercially available assays (18). Typically, the first detectable antibody in human blood is immunoglobulin M (IgM), followed by immunoglobulin G (IgG). IgM is detectable for at least 5 days; these antibodies decline more rapidly, averaging less than two and a half months, and are associated with a mild-to-moderate clinical course of the disease. Instead, IgG is detectable for at least 14 days; these protective neutralizing antibodies, which are linked to severe clinical outcomes of the disease, remain detectable for at least 4 months (19, 20).

This study fills a research gap by examining how occupational exposure influences viral load and the immunological response to SARS-CoV-2 while also considering its potential role in disease transmission. By comparing essential workers in a municipality of the Balearic Islands to non-essential workers and their children, this study provides insights into how different occupational exposures may contribute to the spread of the virus. Unlike previous studies that focused primarily on the general population or healthcare workers, this research examines a diverse group of essential workers across multiple sectors, offering a broader assessment of occupational risk and its implications for both workers and their families.

## 2 Methods

### 2.1 Study design

A total of 504 participants living in the municipality of Andratx, Mallorca, Balearic Islands, Spain, were included in this epidemiological prospective, longitudinal, and codified study following two cohorts: the essential and non-essential groups. The Essential Collective was followed on five occasions between June 2020 and February 2021 (approximately a monthly follow-up), while the non-essential collective was followed on three occasions (approximately a quarterly follow-up). The sons and daughters of both groups were analyzed on the three occasions indicated (approximately quarterly follow-ups).

The Essential Collective included all workers who carried out crucial tasks in the municipality of Andratx (local police, civil protection, citizens’ attention service, cleaning and maintenance brigade, City Council staff for public attention, and civil guards and workers from health), plus their sons or daughters, if any, and who resided in the municipality of Andratx. The non-essential collective included adults registered in the municipality of Andratx with the same age and sex characteristics as the members of the essential group (matched samples), randomly selected from among the adults registered in this municipality, plus their sons or daughters if there were any.

Participants were chosen based on the following inclusion criteria: (1) Adults: those aged 18–65 years, essential workers in the municipality of Andratx (essential group) and adults of the same age registered with Andratx City Council (non-essential group). (2) Children and teenagers aged 2–18 years, children of essential workers, or adults registered with Andratx City Council. The following exclusion criteria were utilized: Inability or unwillingness to give informed consent, inability to follow scheduled visits, serious psychiatric disorders, severe conditions with at least 24 months of life expectancy, immunodeficiency cases, and addiction to illicit drugs.

Participants were apprised of the trial’s purpose and potential consequences, and each provided written consent to partake. The study adhered to the ethical guidelines outlined in the Declaration of Helsinki, and the experimental protocol received approval from the Balearic Islands Ethics Committee (ref. IB 4276/20 PI; approval date: 22 December 2020).

**Figure 1:**
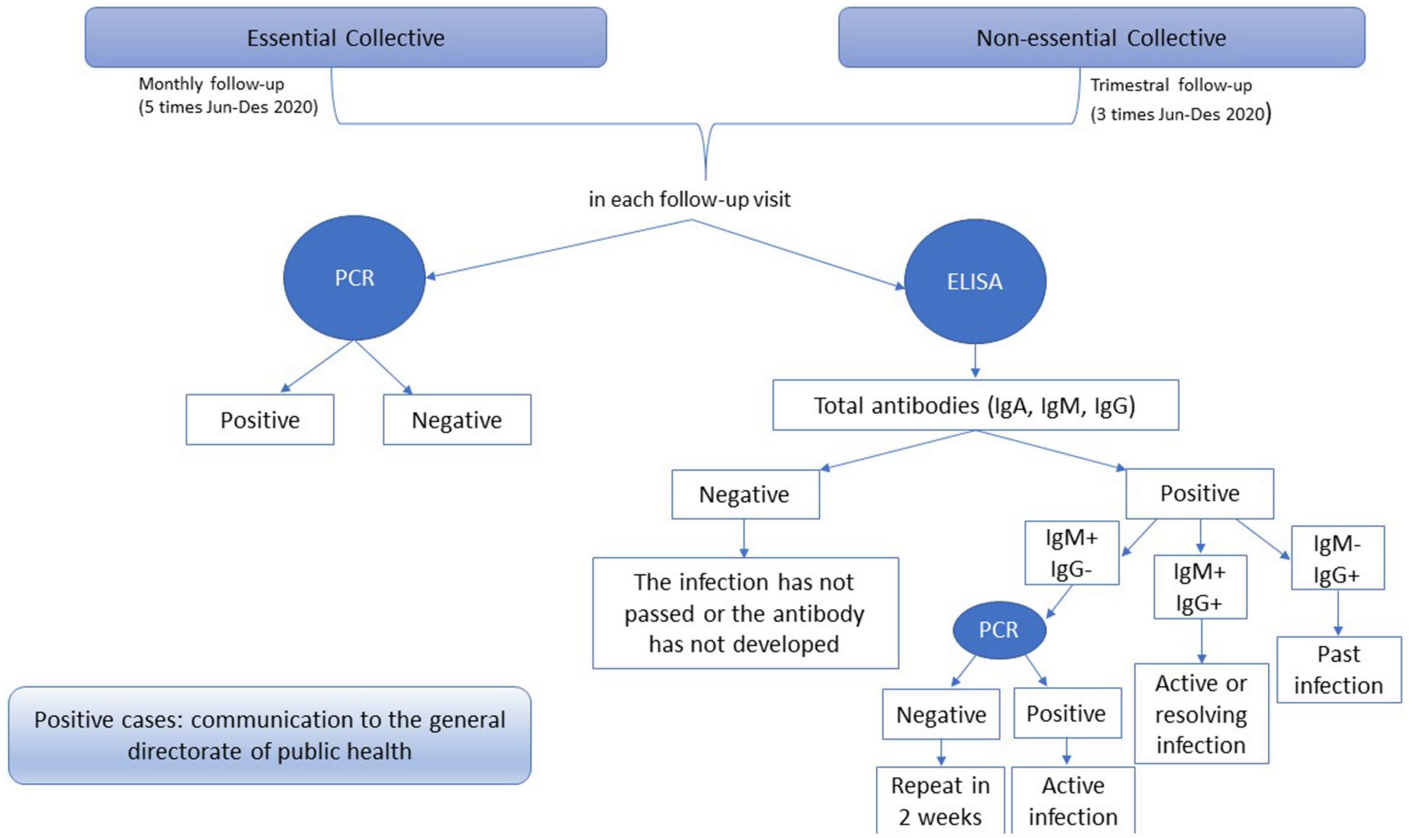
Flowchart of the study.

At each follow-up visit, nasopharyngeal exudate swab samples were collected for the detection of SARS-CoV-2 (COVID-19) by polymerase chain reaction (PCR) and blood samples for isolated plasma for the determination of total antibodies using ELISA kits. If the result was positive for total antibodies, the determination of immunoglobulin M (IgM) and immunoglobulin G (IgG) antibodies was carried out. On the one hand, if IgM was positive and IgG was negative, a PCR was carried out to confirm whether the infection was active. On the other hand, if both immunoglobulins were positive, the infection was active or resolved. Finally, if IgM was negative and IgG was positive, it meant a past infection. The flowchart of the study is shown in Figure 1.

### 2.2 Recruitment

The participants of the study were recruited by the staff of the Andratx City Council via e-mail or by telephone. Participants are informed by e-mail or telephone about the study at the first visit (recruitment), sending them the information sheet to the participants and the informed consent for their signature if they decide to participate in the study, as well as a battery of frequently asked questions. Only after the delivery of the signed consent, was the questionnaire filled out on their socio-demographic characteristics and state of health, as well as the taking of serological samples and smears of nasopharyngeal exudate in facilities owned by Andratx City Council arranged for that purpose (Basic Health Unit of municipality of Andratx, at that time closed for the usual queries).

### 2.3 Sociodemographic characteristics

Sociodemographic characteristics were collected, including sex (male/female), age (years), identification of area-collective (essential/non-essential), municipality of residence (indicate), and primary state of health by questionnaires.

### 2.4 Nasopharyngeal exudate smear collection

Doctors and nurses collected the nasopharyngeal exudate smear after previous training and were dressed in personal protective equipment (PPE). Sterile kits were used in individual sample collection tube containers with an inactivated viral transport medium (Ref. DW-80005-AB, Quimigen, CliniSciences Group). Briefly, the sample was taken with a fine and flexible swab, which was first introduced through one nostril to the nasopharynx and then through the other. The swab reached a depth equal to the distance between the nostrils to the external opening of the ear. The swab was left in place for several seconds to absorb secretions. The swab was slowly removed while rotating. Two or three 180° rotations were made and kept in contact with the mucosa for 5 s. Swabs were immediately inserted into sterile tubes containing 2–3 mL of viral transport medium.

### 2.5 Blood sample collection and processing

Blood samples were collected from the antecubital vein using vacutainers containing ethylenediaminetetraacetic acid (EDTA) as an anticoagulant by trained professionals protected by PPE. Participants and professionals followed all the corresponding safety rules during the blood extraction: patients always wore masks, and healthcare professionals touched the blood tube at no time; a transparent plastic glove always protected them. The blood samples were treated as potentially infectious, category B, and transported in triple packaging, according to UN3373. The laboratory professionals also received prior training and were protected with PPE during the reception of the samples, the plasma extraction procedure, and all the determinations. Plasma samples were acquired through blood centrifugation at 1700 x *g* for 15 min at 4°C.

### 2.6 Detection of viral load by real-time polymerase chain reaction (PCR)

The detection of SARS-CoV-2 was carried out using the LightMix® Modular SARS-CoV-2 (COVID-19) E-gene (Ref. 53-0776-96, TIB MOLBIOL, Berlin, Germany), following the manufacturing instructions, with negative and positive controls, with a sensitivity of 97.9%. The LightCycler® 480 Instrument was used to perform the PCR process in a series of sequential steps. It begins with the reverse transcription of viral RNA into complementary DNA. The next step involves denaturation, where the sample is heated to separate DNA strands and activate the enzyme. This is followed by the cycling phase, in which PCR amplification occurs through repeated heating and cooling cycles. Finally, the process concludes with a cooling step to stabilize the instrument and the amplified DNA.

### 2.7 Detection of antibodies by ELISA kits

The immunoassay kit (Ref. EU3125 Fine Test, Wuhan, China) was used to qualitatively determine 2019-nCoV(N)-Ig antibodies in plasma (total antibodies of SARS-CoV-2), adhering to the manufacturer’s guidelines; according to the manufacturer, the cutoff value was “mean absorbance of negative control (NCx)” multiplied by 2.1. The measure of antibodies IgM (Ref. EH4940) and IgG (Ref. EH4397) was performed with a quantitative ELISA kit (Fine Test, Wuhan, China), following the manufacturing instructions. The range of the human anti-SARS-CoV-2 (N) IgM ELISA kit was 0.781–50 ng/mL, and the sensitivity of the kit was 0.469 ng/mL. Regarding the human anti-SARS-CoV-2 (N) IgG ELISA Kit, the range was 3.906–250 ng/mL, and the sensitivity of the kit was 2.344 ng/mL. A standard curve was conducted to determine the concentration of IgG and IgM, carrying out successive dilutions from standard tubes (1:2, 1:4, 1:8, 1:16, 1:32, and 1:64).

### 2.8 Additional security considerations

All the necessary precautions were taken when handling samples from potential COVID-19 patients. These included the use of personal protective equipment (FFP2 masks, gowns, gloves, and eye protection), strict hygiene measures, hydroalcoholic gel, single-use laboratory materials, and specialized containers for infectious substances. Additional safety protocols ensured proper sample handling, spatial distancing, minimized contact duration, and the use of class II biological safety cabinets and centrifuges with safety covers.

### 2.9 Statistical analysis

#### 2.9.1 Software and tools

Statistical analysis was conducted using the Statistical Package for the Social Sciences (SPSS, v.28 for Windows, IBM Software Group, Chicago, IL, USA).

#### 2.9.2 Categorical variables

The chi-square test was used to analyze categorical variables, which are presented as sample size and percentage.

#### 2.9.3 Continuous variables

Before analysis, the normal distribution of continuous variables was assessed. Variables following a normal distribution, expressed as mean and standard deviation (SD), were analyzed using Student’s *t*-test.

#### 2.9.4 Significance threshold

A *p* < 0.05 was considered indicative of a statistically significant difference.

## 3 Results

Table 1 shows the population distribution in this study, which was classified by sex and age; no significant differences were reported between the populations.

**Table 1:** Distribution of the population analyzed by sex and age.

	Total	2–10	11–20	21–40	41–60	<60
	*n* (%)	*n* (%)	*n* (%)	*n* (%)	*n* (%)	*n* (%)
Total	504 (100)	47 (9.3)	42 (8.3)	145 (28.8)	228 (45.2)	42 (8.3)
Women	268 (53.2)	22 (8.2)	19 (7.1)	83 (31.0)	123 (45.9)	21 (7.8)
Men	236 (46.8)	25 (10.6)	23 (9.7)	62 (26.3)	105 (44.5)	21 (8.9)

There were no statistical differences between the populations; populations were homogenous when sex and age were considered (*p* = 0.555 for the chi-square test).

Table 2 shows the population distribution analyzed by essential or non-essential workers; statistical differences were observed between essential and non-essential workers. Table 3 represents the distribution of essential workers analyzed by sex; statistical differences were detected between essential workers. Table 4 shows participation in scheduled sessions: Sessions 1, 3, and 5 were for the whole population, whereas sessions 2 and 4 were for essential workers. Session 1 was from 14 to 28 July 2020; session 2 was from 28 to 30 September 2020; session 3 was from 9 to 25 November 2020; session 4 was from 15 to 17 December 2020; and session 5 was from 1 to 17 February 2021. The participation of essential workers between sessions 2 and 4 was similar. In sessions 1, 3, and 5, participation decreased a bit between sessions 1 and 5.

**Table 2:** Distribution of the population analyzed by essential or non-essential workers.

	Total	Essential workers	Essential workers’ sons	Non-essential workers	Non-essential workers’ sons
	*n* (%)	*n* (%)	*n* (%)	*n* (%)	*n* (%)
Total	504 (100)	157 (31.2)*	25 (5.0)	266 (52.8)*	56 (11.1)
Women	268 (53.2)	69 (25.7)*	12 (4.5)	163 (60.8)	24 (9.0)
Men	236 (46.8)	88 (37.3)*	13 (5.5)	103 (43.6)*	32 (13.6)

*There were statistical differences between essential and non-essential workers (*p* = 0.002; for the chi-square test).

**Table 3:** Distribution of essential workers analyzed by sex.

	Local police	Civil protection	Citizens’ Attention	Cleaning and maintenance brigade	Social services	Civil guard	Health center	Others
	*n* (%)	*n* (%)	*n* (%)	*n* (%)	*n* (%)	*n* (%)	*n* (%)	*n* (%)
Total	28 (18.4)	18 (11.8)	9 (5.9)	13 (8.6)	18 (11.8)	14 (9.2)	26 (17.1)	26 (17.1)
Women	1 (1.5)	7 (10.3)	6 (8.8)	2 (2.9)	16 (23.5)	2 (2.9)	20 (29.4)	14 (20.6)
Men	27 (32.1)	11 (13.1)	3 (3.6)	11 (13.1)	2 (2.9)	12 (14.3)	6 (7.1)	12 (14.3)

There were statistical differences between essential workers (*p* < 0.001 for the chi-square test). Five missed cases. Others were pharmacists and pharmacy workers, other municipal officials, music school, cleaners, school custodians, city council members, library staff, tax management, nursery personnel, laundry staff, supermarket managers, animal shelter staff, restaurant personnel, gardeners, teachers, taxi drivers, and others.

**Table 4:** Participation in scheduled sessions.

	Total (*n*)	Women (*n*)	Men (*n*)
Session 1	504	268	236
Session 2	133	58	75
Session 3	431	231	200
Session 4	127	57	70
Session 5	435	228	207
Total participation	1630	842	788

Sessions 1, 3, and 5 were for the whole population, whereas sessions 2 and 4 were for essential workers. Session 1: from 14 to 28 July 2020; session 2: from 28 to 30 September 2020; session 3: from 9 to 25 November 2020; session 4: from 15 to 17 December 2020; session 5: from 1 to 17 February 2021.

The PCR test results are shown in Table 5. The RT-PCR analyses showed a low infection rate among the inhabitants of the municipality of Andratx almost since the start of the pandemic. Even so, a positive case was found when screening the entire general population, specifically, a woman.

**Table 5:** Results of positivity and negativity of infection by COVID-19 in the nasopharynx and oropharynx smears (PCR test).

	Total (*n*)	Women (*n*)	Men (*n*)
Session 1			
Positive PCR	0	0	0
Negative PCR	504	268	236
Session 2			
Positive PCR	0	0	0
Negative PCR	133	58	75
Session 3			
Positive PCR	1	1	0
Negative PCR	431	231	200
Session 4			
Positive PCR	0	0	0
Negative PCR	127	57	70
Session 5			
Positive PCR	0	0	0
Negative PCR	435	228	207
Total PCRs	1,630	842	788

Table 6 shows the total antibodies of SARS-CoV-2 [2019-nCoV(N)-Ig antibody] resulted in plasma samples stratified by essential workers, essential workers’ sons, non-essential workers, and non-essential workers’ sons. Significant differences were observed in session 3 between the four groups, among the vital workers and the general population, and between children of essential workers and children of the general population. Positive cases of total immunoglobulins increased over time, especially in session 5, from 1 to 17 February 2021.

**Table 6:** Results of total antibodies of SARS-CoV-2 in plasma samples stratified by essential workers, essential workers’ sons, non-essential workers, and non-essential workers’ sons.

	Essential workers	Essential workers’ sons	Non-essential workers	Non-essential Workers’ sons	*p*-value
	*n* (%)	*n* (%)	*n* (%)	*n* (%)	
Session 1 (*n* = 504)					
+	3 (1.9)	0 (0.0)	0 (0.0)	0 (0.0)	0.086^1^
−	145 (98.1)	25 (100)	262 (100)	56 (100)	0.025^2^
Session 2 (*n* = 133)					
+	8 (6.0)	–	–	–	–
−	125 (94.0)	–	–	–	–
Session 3 (*n* = 428)					
+	8 (5.8)	1 (4.8)	5 (2.2)	1 (2.2)	0.036^1^
−	129 (94.2)	20 (95.2)	221 (97.8)	44 (97.7)	0.036^2^ 0.036^3^
Session 4 (*n* = 127)					
+	10 (7.9)	–	–	–	–
−	117 (92.1)	–	–	–	
Session 5 (*n* = 432)					
+	10 (6.8)	2 (9.5)	16 (7.3)	1 (2.2)	0.328
−	137 (93.2)	19 (90.5)	203 (92.7)	44 (97.8)	0.977 0.050

The percentages represent the proportion that these people represent within each collective *Statistical differences for the chi-square test: ^1^among the four groups; ^2^essential workers vs. general population; ^3^children of essential workers vs. children of the general population.

**Figure 2:**
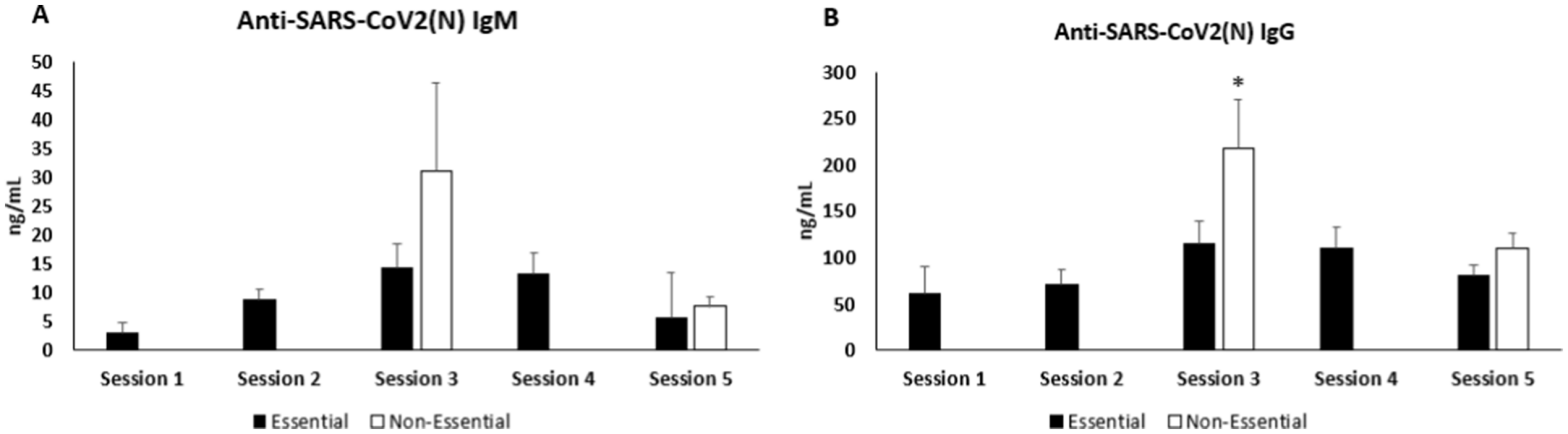
Anti-SARS-CoV-2(N) determinations. **(A)** IgM levels. **(B)** IgG levels. Statistical analysis: student’s *t*-test. *Significant differences vs. essential, *p* < 0.05.

The anti-SARS-CoV-2 (N) IgM levels are shown in Figure 2A. Like anti-SARS-CoV-2(N) IgG, in session 1 only, anti-SARS-CoV-2(N) IgM in essential workers with positive total immunoglobulins was due to none of the non-essential workers having positive total antibodies of SARS-CoV-2. In session 2, levels of anti-SARS-CoV-2(N) IgM also appeared to increase compared to session 1. In session 3, in November, non-essential participants presented higher levels of anti-SARS-CoV-2 (N) IgM than essential ones. In session 4, the levels of anti-SARS-CoV-2(N) IgM seemed to be about the same as in session 3. Finally, in the last session, session 5, the anti-SARS-CoV-2(N) IgM levels reduced in both groups compared to session 3.

The anti-SARS-CoV-2(N) IgG levels are shown in Figure 2B. In session 1, anti-SARS-CoV-2(N) IgG levels were determined only in essential workers with positive total immunoglobulins because none of the non-essential workers had positive total antibodies of SARS-CoV-2. In session 2, essential workers showed a slight increase in anti-SARS-CoV-2 (N) IgG levels compared to session 1. In session 3, a significant rise in anti-SARS-CoV-2 (N) IgG levels was observed in non-essential workers and essential workers. In session 4, a small increment was observed in anti-SARS-CoV-2 (N) IgG levels in essential workers concerning session 2. In session 5, the anti-SARS-CoV-2 (N) IgG levels decreased in both groups compared to session 3.

## 4 Discussion

This SARS-CoV-2 (COVID-19) screening study provides valuable insights into the virus’s progression from June 2020 to February 2021 in Andratx, Mallorca. By analyzing both essential and non-essential workers, along with their children, the study offers a comprehensive assessment of occupational exposure and its impact on viral spread and immune response (21). The homogeneity of the sample distribution by sex and age strengthens the reliability of the findings.

A previous study reported seven factors for exposure to the COVID-19 virus: severe COVID-19, loss of income, limited access to essentials, disruptions to activities, disruptions to living conditions, and designation as an essential worker. It also reported five factors for impact: self and family relationships, physical wellbeing, emotional wellbeing, social wellbeing, and distress (22).

A key observation was the low overall infection rate, as indicated by RT-PCR results. Only one positive case was identified, which could have helped reduce the spread of the disease in the core of S’Arracó (Andratx, Mallorca), which could have affected hundreds of people. This finding suggests that public health measures in this place were effective at limiting virus transmission. RT-PCR has better accuracy than quick tests and is considered the gold standard test for detecting SARS-CoV-2. Even in the first days of infection, even before the onset of symptoms (23), as in the case of this study, who was an asymptomatic positive person. However, its high cost and the need for specialized laboratory infrastructure remain challenges (23).

Serological data further support the effectiveness of containment measures. The study observed an increase in anti-SARS-CoV-2 (N) IgM and IgG levels, particularly in non-essential workers, during session 3 (November 2020). This trend coincides with a relaxation of COVID-19 restrictions during the summer of 2020, leading to increased exposure and subsequent immune responses when the Plan for the Transition to a New Normality was approved (24). However, the increase in cases in the population was not reflected in session 2 of September, since the subjects analyzed were essential workers, including health professionals, which demonstrates the effectiveness of the use of masks (25).

Moreover, the increase observed in November 2020 as reflected in this study, also took place in Spain and across most European countries. An increasing trend was recorded in the number of cases, with incidents that placed most of the territory at a high or very high-risk level according to international standards and the national ones established in the Coordinated Response Actions document to control the transmission of COVID-19, approved in the plenary session of the Interterritorial Council of the National Health System on 22 October 2020 (26). Furthermore, on 27 November 2020, there was an update of the limitations to contain the spread of infections caused by SARS-CoV-2, specifically the limitations on the freedom of movement of people at night, limitations on the entry and exit of people from the territory, limitations on the permanence of groups of people in public and private spaces, and limitations on the permanence of people in places of worship, detailing each limitation depending on the level of health alert (0, 1, 2, 3, and 4) (27).

In line with the restrictions in place in December 2020 in the Spanish autonomous communities and cities and the special circumstances typically arising during the Christmas holidays 2020 regarding social gatherings, it was considered to limit gatherings of family and close friends to a maximum of 10 people, except for cohabitants, to sufficiently reduce the risk of virus transmission while allowing for the social life traditionally associated with these festivities (28). Furthermore, as a limitation of night mobility, it is worth highlighting a curfew at 1:30 a.m. on 24 and 31 December 2020 (28).

Moreover, on 30 December 2020, vaccination began in the Balearic Islands, with Pfizer, Moderna, or AstraZeneca vaccines against SARS-CoV-2. Receiving a single dose of either the Pfizer-BioNTech or Oxford AstraZeneca vaccines significantly reduced symptomatic COVID-19 in older adults and provided additional protection against severe illness (29, 30).

Both vaccines demonstrated comparable effectiveness, with protection sustained throughout the follow-up period of over 6 weeks. Furthermore, a second dose of Pfizer-BioNTech offered even greater protection against symptomatic disease (31).

The study’s findings align with prior research indicating that IgG antibodies persist longer than IgM, providing prolonged protection against reinfection. This observation underscores the importance of vaccination in sustaining immunity beyond the natural antibody response. The data suggest that individuals who had prior exposure to SARS-CoV-2 benefited from both natural and vaccine-induced immunity, reinforcing the need for continued public health efforts to promote vaccination (32).

### 4.1 Strengths and limitations

This study has several strengths. First, it is a prospective cohort study that allows for a longitudinal assessment of SARS-CoV-2 infection and immune response over time. The study design includes both essential and non-essential workers, as well as their children, providing valuable insights into occupational exposure and household transmission dynamics. Additionally, the study uses gold-standard diagnostic techniques, such as RT-PCR testing and ELISA-based serological assessments, ensuring high accuracy in detecting infections and immune responses. Furthermore, the inclusion of a well-defined population from the municipality of Andratx, Mallorca, ensures a focused analysis of local transmission and the effectiveness of public health measures. Finally, adherence to rigorous ethical standards and methodological consistency strengthens the reliability and validity of the findings. Despite its strengths, this study has some limitations. First, the sample size, while sufficient for local analysis, may limit the generalizability of the findings to larger populations or different geographical areas. Second, the study focuses on a specific time frame (June 2020 to February 2021), meaning that evolving variants of SARS-CoV-2 and subsequent vaccination campaigns may have influenced infection rates and immune responses differently in later phases of the pandemic.

### 4.2 Implications for future research

Future studies should consider expanding the sample size and including diverse geographical locations to enhance the generalizability of the findings. Long-term follow-up studies could provide insights into the durability of immunity and the impact of emerging variants on infection rates. Additionally, studies incorporating genomic sequencing could help identify potential differences in viral strains affecting essential and non-essential workers. Finally, a more detailed investigation of behavioral factors, such as adherence to public health measures and social interactions, would provide a more comprehensive understanding of transmission dynamics and the effectiveness of different preventive strategies.

## 5 Conclusion

The effectiveness of COVID-19 safety measures depends on their comprehensive and coordinated implementation. While each measure individually contributes to reducing transmission, their combined application has a synergistic effect that significantly enhances its overall effectiveness. Public adherence and continuous adaptation of these measures in response to evolving scientific evidence and virus variants are critical to sustaining control over the pandemic. Therefore, the safety measures taken to control the COVID-19 pandemic in Andratx, Mallorca, Spain, were efficient.

Overall, this study highlights the importance of timely public health interventions in controlling the SARS-CoV-2 spread. The effectiveness of safety measures, particularly among essential workers, demonstrates the role of PPE and workplace protocols in minimizing infection risk. Future research should explore the long-term durability of immunity, the impact of emerging variants, and the interplay between natural infection and vaccination in maintaining population-wide protection.

## Data Availability

The raw data supporting the conclusions of this article will be made available by the authors, without undue reservation.
